# Indigenously developed digital handheld Android-based Geographic Information System (GIS)-tagged tablets (TABs) in malaria elimination programme in Mangaluru city, Karnataka, India

**DOI:** 10.1186/s12936-019-3080-8

**Published:** 2019-12-26

**Authors:** B. Shantharam Baliga, Animesh Jain, Naren Koduvattat, B. G. Prakash Kumar, Manu Kumar, Arun Kumar, Susanta K. Ghosh

**Affiliations:** 1 Kasturba Medical College, Manipal Academy of Higher Education, Mangalore, Karnataka 575003 India; 2 I-Point Consulting, Punja Arcade, Lalbagh, Mangalore, Karnataka 575003 India; 3 Directorate of Health and Family Welfare Services, Government of Karnataka, Bangalore, Karnataka 560009 India; 4 City Corporation, Lalbagh, Mangalore, Karnataka 575003 India; 5 Department of Public Health, Dakshina Kannada District, Mangalore, Karnataka 575001 India; 6 ICMR-National Institute of Malaria Research (Field Unit), Nirmal Bhawan–ICMR Campus, Poojanahalli, Kannamangla Post, Devanahalli Taluk, Bangalore, Karnataka 562110 India

**Keywords:** Malaria, TABs, Mobile app, GIS, Software, Information technology, Digitization, Smart surveillance, Incidence-centric, Malaria elimination, Mangaluru

## Abstract

**Background:**

Under-reporting, delayed diagnosis, incomplete treatment and inadequate vector management are few among many factors responsible for uninterrupted transmission of malaria in India. Information technology (IT) and mobile apps can be utilized effectively to overcome these hurdles. Indigenously developed digital handheld geographic information system (GIS)-tagged Android-based tablets (TABs) has been designed especially for implementation of digitization protocol. This has changed the effectiveness of malaria surveillance and intervention strategies in a malaria endemic area of Mangaluru city, Karnataka, India.

**Methods:**

A software was developed and implemented for control measures to create a digital database of each malaria case. Secondary data analyses were carried out to determine and compare differences in malariometric indices between pre- and post-digitization years. With the introduction of this software active surveillance, information education and communication (IEC), and anti-vector measures were made ‘incidence-centric’. This means that the entire control measures were carried out in the houses where the malaria cases (index cases) were reported and also in surrounding houses.

**Results:**

Annual blood examination rate (ABER) increased from 13.82 to 32.8%. Prompt reporting of new cases had improved (36% within 24 h and 80% within 72 h). Complete treatment and parasite clearance time were documented in 98% of cases. In the second post-digitization year untraceable cases reduced from 11.3 to 2.7%; contact blood smears collection also increased significantly (*p *< 0.001); Slide Positivity Rate (SPR) decreased from 15.5 to 10.48%; malaria cases reduced by 30%.

**Conclusions:**

IT is very useful in translation of digitized surveillance to core interventions thereby effectively reduce incidence of malaria. This technology can be used effectively to translate smart surveillance to core interventions following the ‘1-3-7-14’ strategy.

## Background

India is a signatory to the National Framework of Malaria Elimination (NFME) with a goal to eliminate malaria by 2030 [1]. Global technical strategy (GTS) of the World Health Organization (WHO) has recommended a 3-pillar transforming surveillance into core interventions for malaria elimination [2]. However under-reporting, delayed diagnosis, incomplete treatment and inadequate vector management are few among many factors responsible for uninterrupted transmission of malaria in India [2, 3]. Currently, malaria surveillance is the weakest in countries with highest malaria burden, rendering it difficult to accurately assess disease trends and plan interventions. Only about one-tenth of the estimated 198 million cases that occurred in 2013 were detected and reported through national malaria surveillance systems (WHO’s uncertainty range for malaria cases is 124 to 283 million) [4, 5]. Even there is a huge disparity of malaria burden in India. In 2017, the WHO reported an estimated 219 million malaria cases and 435,000 related deaths in the world where India’s contribution was 4% with 9.6 million cases and 16,723 deaths. In contrast, National Vector Borne Disease Control Programme (NVBDCP) reported 0.84 million malaria cases and 174 related deaths. This means there is a serious problem of case reporting system due to poor surveillance in many states [1].

Information technology (IT) and mobile apps can be utilized effectively to overcome these hurdles. IT, in particular, might help by networking all the stakeholders for generating real-time communication of information to implement appropriate and timely intervention measures. Currently, Android-based mobile apps are under process of deployment in the field. An innovative open data kit (ODK)-based Android app named SMART malaria surveillance for malaria case investigation has been developed in Ethiopia. This device uses Google Maps and embedded geo-referenced house census data. Here the surveillance assistants (SAs) obtain all necessary basic information of each malaria case from the outpatient department register book. The SAs create an index case record including the hamlet name after determining the illness criteria. This information helps case investigation in the field [6].

Similarly, in Philippines, an Android web-based online malaria information system (OLMIS) has been developed to serve as a tool for data collection, processing, reporting and use of information contemplating the malaria elimination strategy. The system can work offline also. The health workers can easily record diagnosis and treatment with the apps in hand. The OLMIS system is currently in the final stage of field testing and ready for full implementation [7].

Mangaluru city is situated on the southwestern coast of Karnataka state, India. The local metropolitan area with a population over 0.6 million is endemic for malaria since 1995 [8–10].

*Anopheles stephensi* was incriminated for vector of malaria in this city [11]. There was a boom in construction-related work which was associated with migratory labourers from various parts of the country mostly from malaria endemic states of Odisha, West Bengal, Madhya Pradesh, Jharkhand, Chhattisgarh and states of northeast India. Labourers from other malaria endemic districts from Karnataka also migrated to the city engaged on the construction work. This was one of the main reasons for malaria transmission in the city [12]. Various control programmes at the national level such as Modified Action Plan (MAP) in 1995, National Malaria Eradication Programme (NMEP) in 1999, NVBDCP in 2002, and subsequent modified versions of these control strategies have been implemented in the city from 1995 till 2014. However, these programmes had very variable impacts on the disease in this city which is contributing 70–80% of total malaria cases in Karnataka [1, 10]. *Plasmodium vivax* is the predominant species contributing around 85% of the total malaria cases in the city [10, 12]. The local civic authorities engage multipurpose workers (MPWs) for carrying out work related to all vector borne diseases in the city. They follow work protocols as per the guidelines of NVBDCP. Before launching the digitized protocol, the reporting of cases and the action taken were documented manually. Hence, data retrieval was time consuming leading to frequent delays in surveillance, source reduction, anti-vector activities and follow-up of cases to ensure complete treatment. Moreover, the data were not readily available to be compiled for any specific purpose. The surveillance system was not properly organized and effective. The MPWs were given a target to cover 40 houses in a day with manual formats and guidelines. All deficiencies in implementation of control measures led to a cascading effect with a chain of transmission cycles not being interrupted. There was also a little or no timely action to control and prevent new cases. Moreover, no geographical location of disease source could be made available readily. This meant that no focused source reduction steps could be taken up in a timely manner to eradicate the infection focus in the affected urban areas of Mangaluru. Information technology (IT) and mobile apps were utilized effectively to overcome such hurdles by improving networking of all stakeholders for real-time transfer of information for necessary and timely intervention measures.

The gaps, hurdles and deficiencies in implementation of malaria control programme in Mangaluru city were identified after evaluation of existing programme. In view of these challenges identified, in July 2014 the local civic authorities decided to digitize malaria surveillance and control programme with an aim to enhance timely reporting and focused interventions for better source reduction, complete treatment, and vector control mainly larval breeding habitats.

This article describes the design and implementation of this digitization protocol and presents initial secondary data analyses to determine the impacts in the post-digitization years.

## Methods

### Development of GIS-based software

**Fig. 1:**
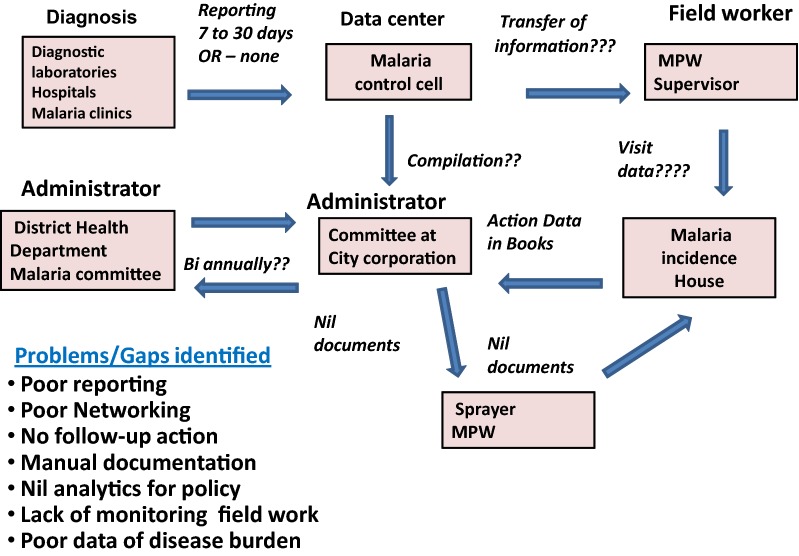
Process and problems/issues identified in anti-malarial programme in the urban area of Mangaluru city

Malaria Control System is a state of the art suite of software product to transform existing programme in the Mangaluru city into a technology driven intelligence-assisted programme. Between August 2014 and September 2015, a software programme was designed for effective micro-implementation of all anti-malarial activities (Fig. 1). At the core, this system is a GIS-tagged mobile app for civic body field force for reporting and monitoring of incident cases as per the NVBDCP guidelines, mosquito breeding site reporting and intervention monitoring [13]. Civic body, geographical and administrative structures are mapped into the software for efficient monitoring, workload allocation and reviews. It has an online (web) app for healthcare providers (hospitals, diagnostic clinics etc.) to report malaria cases, and submit statutory reports. Back end data is automatically generated to excel sheet and analyses were carried out to identify the hot spots, high risk wards, and also fortnightly/monthly trends. Another online (web) app is also linked to analyse effectiveness, identify issues and defaulters, and take timely administrative actions.

**Fig. 2:**
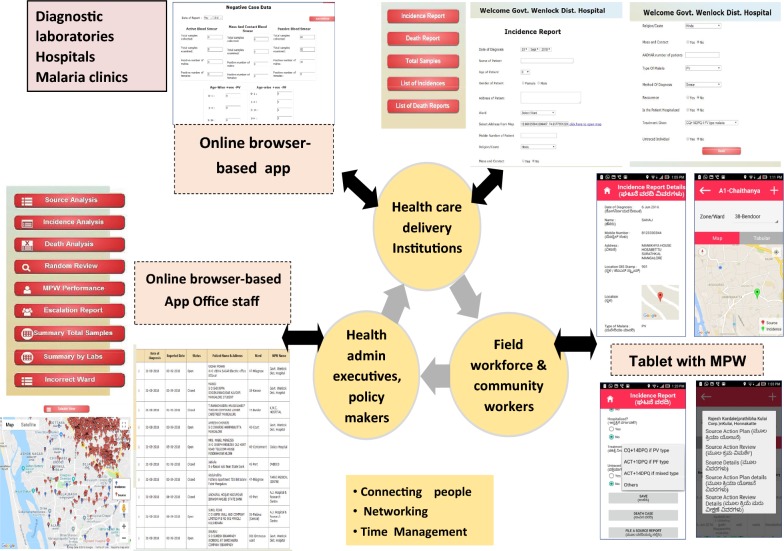
Concept, design and functioning of malaria control software (screenshots of dashboard, Multi Purpose Worker tablets and lab login screen)

Figure 2 describes various components of the software and its functionalities. Important salient features of software are: (a) all stakeholders instantaneously connected soon after case details are entered on the website, thereby translate reporting into field action; (b) facilitates geospatial mapping; (c) mandatory closure of cases, and also interventions of mosquito breeding habitats after effective completion of action; (d) document evidences of field activities and parasite clearance time. Briefly, programme incorporates most strategies for malaria from surveillance to complete treatment along with vector control activities—a distinct viable solution.

### Introduction of digital TABs in malaria surveillance

In the digital India era, most of the health staffs have experience in using Android-based smart phones. This helped in better understanding of the operational procedures of the working systems. Initially, field trials were carried out to study the operational feasibility and troubleshooting along with enhancement of systems. Eight MPWs were given hands-on training in the field during field trials, and these trained MPWs in turn trained other 54 MPWs in the field. In October 2015, the programme was scaled up to cover the entire city of Mangaluru spread over in 60 wards (Fig. 2). Additional training was carried out in the field for the needy ones. Separate workshops and training programmes were conducted for laboratory technologists, MPWs and hospital authorities both in private as well as public health systems for reporting of cases before scaling up of the programme to the entire city. At present the programme is governed and maintained by the local civic authorities.

### Malaria control programme after digitization

Malaria control operations in Mangaluru city were fully digitized in October 2015, and a network between diagnostic centres, field workers (MPWs) and administrators was established. As soon as a case of malaria was diagnosed in the field or any laboratories, hospitals and malaria clinics, assigned personnel were statutorily required to login and enter case information through online web portal into the software. Hospitals and clinics both in public and private health systems began to report malaria cases online. Information on newly reported cases was made available from the TAB devices provided to each MPW. Online entry of new case triggered an alert to these workers on their TABs who in turn mandatorily visited the households, meet patients and their families, ensure complete treatment and survey the neighbourhoods. These TABs are loaded with software/app which provided them information regarding anti-malarial activities to be carried out as per the NVBDCP guidelines. Activities included search for source of mosquito breeding sources, collection of blood smears from any fever case in and around malaria-positive households. MPWs were instructed to record all their visits and activities on their devices. It was mandatory for them to close each malaria positive case and mosquito breeding sources after complete action in the field. GIS-tagged patient information helped the MPWs to visit these houses and record pre-determined information about cases and field activities. The activities and movements of the MPWs were monitored through GIS.

City corporation executives were motivated to use only data from the online system for their reviews and presentations instead of the data from manual records and registers. Currently, 70 field workers (MPWs, Health Supervisors) and 85 health care providers (hospitals, clinics, and diagnostic centres) are using these apps/solutions.

### Secondary data (pre- and post-digitization) analysis

#### Study design

##### Process of digitization

This is an uncontrolled field trial to know the impact of application of digitization software on operational parameters of malaria control as compared with situation before interventions. All cases of malaria reported in the geographical area of Mangaluru Municipal Corporation were included in the study. Cases diagnosed by the local diagnostic labs, hospitals, malaria clinics, and also surveillance teams, by microscopic examination of blood smears, rapid diagnostic test (RDT) kits and quantitative buffy coat (QBC) fluorescent technique were also included.

Between October 2014 and September 2015 digitization year (DY) when software was conceived, codes were written, programme was developed, field trials were conducted and all the activities carried out were manually documented. First year post-digitization is considered as PDY1 (October 2015 to September 2016), and second year post-digitization is represented as PDY2 (October 2016 to September 2017). Post-digitization is the phase when the software was accepted and well utilized by all the stakeholders namely diagnostic laboratories, hospitals, field workers and administrators.

##### Data inputs

Both active and passive surveillance data, case management details, anti-larval activities undertaken, were considered as real-time digital data on the system from September 2015 onwards. Manually compiled data for 1-year period during digitization phase from October 2014 to September 2015, and a year prior was obtained from malaria control cell of city administration.

##### Data analysis

Collected data were analysed as follows:

1. To assess improvement in surveillance reporting time, and field action.
  - *Time taken to report a malaria positive case*. This indicated reporting behaviour of health workers and facilities (laboratories, clinics and hospitals). This was carried out as back end analysis in the first 15 months after implementation.
  - *Closure of cases after complete treatment*. A case is closed after revisit to their residences, and documentation of evidences for post-treatment parasite clearance time. Number of cases closed indicated completion of treatment and negative blood smear post-treatment of active surveillance around reported case (ASARC). Routine and mandated house visits in the houses of the malaria cases; contact blood smears in surrounding households; identification of mosquito breeding sites and their elimination were conducted.
  - Number of contact blood smears collected in fever cases around the reported case.
  - Annual blood examination rate (ABER) as an indicator for effective surveillance.
2. To assess the effectiveness of interventions.
  - *Surveillance and malariometric indices*: Annual Blood Examination Rate (ABER), Annual Parasite Incidence (API), Slide Positivity Rate (SPR), Slide Falciparum Rate (SFR), Percentage of *P. falciparum* cases (Pf%), and total number of cases reported.
  - All indices were compared with available manually collected data of 1 year prior to digitization (Pre-DY).

### Statistical analysis

Data are represented as descriptive data, trends and Chi-square test was applied wherever appropriate using VassarStats (vassarstats.net) analysis tool. Significance values *p *< 0.05 was considered significant.

## Results

Data were analysed captured from the malaria control system software for 2 years after implementation and compared it with manual data (recorded in books and registers in written forms) for 1 year prior to digitization (pre-DY) and also in the digitization year (DY).

**Fig. 3:**
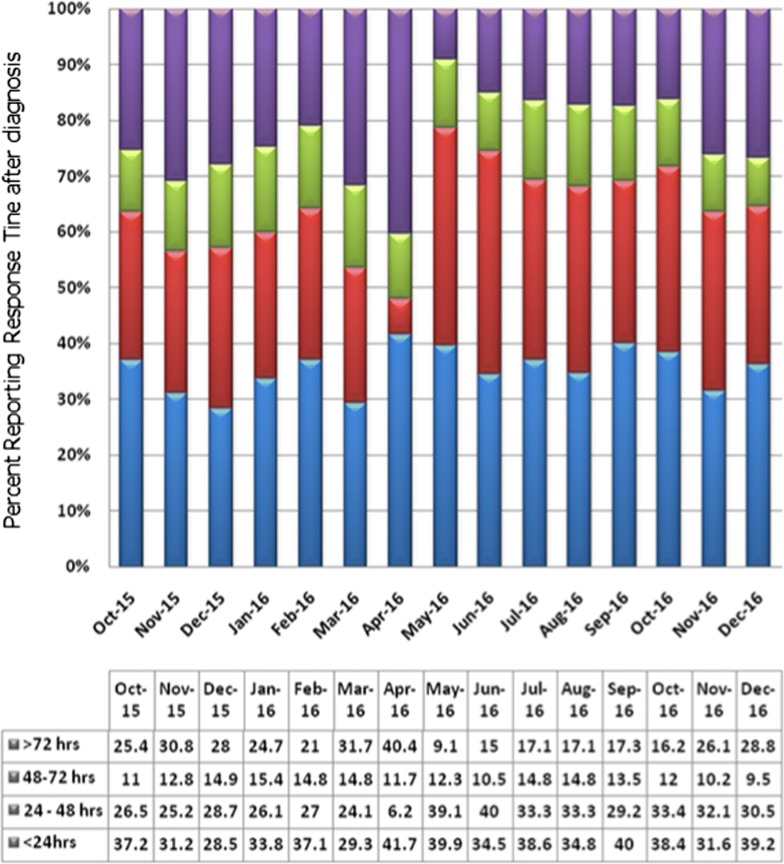
Reporting pattern in relation to time of diagnosis from the point of diagnosis in Mangaluru city

Reporting of cases at the point of diagnosis for their management by MPWs is shown in Fig. 3. Reporting response time was calculated as difference between date and time of diagnosis and reporting on the system. This time-interval was monitored for the first 15 months between October 2015 and December 2016. On an average 36% of cases were reported within 24 h, 80% within 72 h and only 24% of cases were reported later than 72 h. This delay was attributed to the late reporting from smaller centres where intermittent internet services and overburden of work on some serving staff. Continued education and motivation are underway to make more efficient reporting system.

Number of cases wherein complete treatment was ensured, parasite clearance time was documented, and closure of each case is shown in Table 1. Closure of cases was around 80% in the initial months after introduction of the software, which increased to 98% in subsequent months. In first year of digitization 11.3% of cases were untraceable which declined to 2.7% in the second year (*p *< 0.001).

**Table 1:** Month wise distribution of cases, status of cases and closure of malaria cases after complete treatment and proven parasite clearance—as reported on the system

Month	Total cases on the system (no)	Outside city administration limits (no)	Open cases (no)	Open cases in the city (no)	Cases within the limits of civic body administration (no)	Closed cases (no)	Percentage of closure (%)
October 2015	795	7	7	0	788	762	96.70
November	1435	53	317	264	1382	1118	80.89
December	1694	29	323	294	1665	1371	82.34
January, 2016	1144	23	243	220	1121	901	80.37
February	790	18	159	141	772	631	81.73
March	596	50	131	180	546	465	85.10
April	624	146	172	26	478	452	94.56
May	609	120	133	13	489	476	97.34
June	1562	334	418	84	1228	1144	93.15
July	2563	437	630	193	2126	1933	90.92
August	2421	433	445	12	1998	1976	98.89
September, 2016	1289	247	248	1	1042	1041	99.90
Years total	15,522	1897	3226	1428	13,635^a^	11,229	89.27
October 2016	907	164	167	3	743	740	99.59
November	828	150	149	1	678	678	100.00
December	553	76	74	0	477	477	100.00
January, 2017	471	56	57	1	415	414	99.75
February	410	56	56	0	354	354	100.00
March	358	52	57	0	306	301	98.36
April	442	62	66	4	380	376	98.90
May	453	82	82	0	371	371	100.00
June	848	135	145	10	713	703	98.58
July	1542	279	314	35	1263	1210	95.80
August	1771	354	418	64	1417	1353	95.48
September, 2017	1146	235	313	78	911	833	91.43
Years total	9729	1701	1898	196	8028^a^	7810	98.16

^a^Original data from the software includes dual entries does not reflect actual incidence within the city

After the introduction of TABs, active surveillance performed by MPWs was documented appropriately and digitally. A total of 195,009 and 176,389 house-visits were carried out during the first and second years, respectively. These visits were ascertained by GIS tracking. Active surveillance was linked to case reported namely ASARC. Surveillance included complete treatment of case, identification of mosquito breeding habitats followed by action, contact blood smears of fever cases in the houses surrounding each malaria positive case. Non-compliant cases were reported on the system as evidenced. These numbers were 11,337 in the first year and 10,545 in the second year, respectively. These non-compliant cases were closed after revisits and action taken i.e. elimination/chemical treatment/draining of water of each mosquito breeding habitat.

Using the digital TABs, database of water bodies was created mainly targeting wells. A total number of 6261 open wells were identified in 12,397 households for biological anti-larval measures with introduction of guppy fish (*Poecilia reticulata*).

**Fig. 4:**
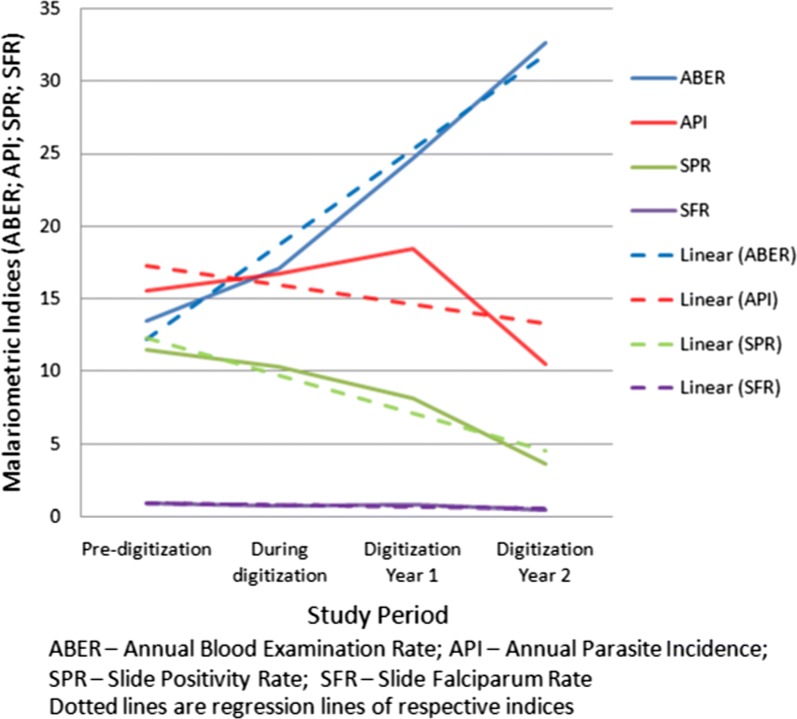
Trends in malaria indices at Mangaluru city pre-, during and post-digitization periods

Impact of improved reporting, surveillance and monitoring subsequent to digitization, and vector control on incidence of malaria, are provided in Tables 2 and 3, respectively. Two years after digitization malaria incidences had reduced by 30.3% when compared to the pre-digitization period. Malaria indices are presented in Fig. 4. At present software is in use and cumulative reduction in incidence of malaria is 67% till date as against in the pre-digitization year (*p *< 0.001). ABER increased from 13.48 to 32.68% (*r*^2^ = 0.950); API decreased to 10.48 from 15.51 (*r*^2^ = − 0.704); SPR from 11.5 to 3.65% (*r*^2^ = − 0.740) and SFR from 0.92 to 0.49% (*r*^2^ = − 0.103).

**Table 2:** Malaria cases reported in the city of Mangaluru in the pre- during and post digitization years

Activities	Pre-DY	Digitization year (DY)	1st year post digitization (PDY1)	2nd year post digitization (PDY2)
Number of slides examined	84,102	106,885	154,409	203,894
Number of contact smears	^a^	^a^	16,541	32,390
Annual blood examination rate	13.82	17.13	24.75	32.68
Slide positivity rate	11.5	10.36	8.17	3.65
Total malarial cases (no)^b^	8867	10,962	12,614	7640
Vivax malaria (no)	8092	10,196	11,277	6245
Falciparum malaria (no)	775	766	1337	1395
	Chi-square trend 164.5		p value < 0.001	

^a^Separate data on blood smear collected not available from fever cases during active surveillance

^b^Compiled after deletions of duplicate cases and cases from outside the corporation administrative limits

**Table 3:** Vector control activities in the city of Mangaluru in the post-digitization years

Activities	1st year post digitization (PDY1)	2nd year post digitization (PDY2)
Number of houses visited	1, 95,009	1,76,398
Source reported	11,337	10,545
Closure of reported sources (%)	89.2	98.1
Number of construction sites with larval breeding	4409	4098
Anti larval action carried out at construction sites	4036 (91.5%)	3837 (93.6%)
Mosquitogenic houses (no)	5527	5817
Anti-larval action—residential	5455 (98.5%)	5676 (97.2%)

## Discussion

At Mangaluru, following digitization, there was noteworthy change in ‘timely reporting’ behaviour of diagnosticians at the point of diagnosis and may be attributed to ease of reporting (Fig. 3). This led to the change in strategies from Early Detection and Prompt Treatment (EDPT) to Early Reporting for Complete Solutions (ERCS). ERCS includes treatment, contact blood smears (active surveillance), anti-vector measures and IEC. Time available for effective field activities also increased as a consequence of digital documentations.

Timing of interventions to break the transmission cycle is important and ideally any new malaria case is required to be investigated within 7 days to reduce transmission risk [14]. Subsequent to digitization, MPWs did work within the transmission period in the houses surrounding malaria cases. Instantaneous transfer of information of newly diagnosed cases to field workforce not only helped in complete treatment but also activated anti-larval/anti-mosquito measures. Case investigation was coupled with IEC in the neighbouring houses following NVBDCP guidelines. Better micro management was observed among the empowered and motivated field workers.

After digitization, approach to control programme was made ‘incidence–centric’ i.e., ASARC. Active surveillance, contact blood smears, IEC, anti-vector measures in and around malaria positive houses were components of ASARC. Similar approach was adopted in Botswana had resulted in drastic reduction of malaria cases between 2012 and 2014 [15]. Given the enormous burden of the disease, ASARC may represent a ‘low hanging fruit strategy’ that means a relatively simpler step with larger gains in disease elimination (Fig. 4).

Improved surveillance and follow-up action has been successful in Sri Lanka which is declared free of indigenous cases of malaria [16]. Active Surveillance is an important component in malaria control activities to reduce parasite load and the MPWs were made mandatory to visit houses to identify fever cases for blood smear examination. ‘Activated passive case detection’ or APCD, a form of passive case detection method adopted in Sri Lanka. This is a system of dedicated malaria-only screening units in public health facilities. Reactive case detection (RCAD) a similar approach was successfully adopted in China. This system and RACD followed in China establish the need for aggressive passive surveillance/contact blood smears [14]. A ‘1-3-7’ strategy was adopted that promoted the elimination process with zero indigenous malaria cases from 2017. This means that strategic action was implemented from diagnosis to treatment within 3 days and public health responses to vector management within day 7 of the case detection. After digitization there was significant increase in active blood smear collection from contacts of newly diagnosed malaria case in Mangaluru city. Here, a strategic action plan of ‘1-3-7-14’ has been envisaged; diagnosis of malaria cases and treatment within 3 days, post-treatment follow-up and public health responses including vector control and contact blood smear surveys by day 7. In case of *P. vivax* infection completion of radical treatment with primaquine within day 14.

Key features of better micromanagement were made mandatory for closure of each case. These features in the software ensured follow-up of patients, collection of contact blood smears and vector control activities. Previously there was no proper documentation of parasite clearance time, although programme emphasized it. Time between reporting and closure of malaria cases was a tool for monitoring field work, and establish accountability in the field. On the contrary, earlier manual system did not emphasize on time dependent interventions. In RACD household members, neighbours and other contacts of passively detected malaria cases are screened for infection, and are treated with effective antimalarial drugs [17].

Second key feature of the software is documentation of closure of reported breeding sites after required remedial action which resulted in effective implementation of anti- larval measures in the field. Elimination of breeding was achieved in 98% of reported sites in the second year.

Penal action on defaulters using digitized data enhanced compliance at construction sites following the local municipality by-laws and also guidelines of NVBDCP. At the back end visits on day 1 and closure of cases on day 14 were also supervised. Penal notices were served and fines were collected from the construction owners when repeated non-compliance of vector control measures by them. Subsequently, the anti-larval measures were undertaken by the construction community improved. Using the software database of large fresh water bodies namely open wells was generated and biological control measures (guppy fish) were taken which also contributed in reducing spread of malaria. Such a large-scale activity is possible due to GIS-tagged tracking, easy availability and accessibility of information on large water bodies. Introduction of fish is an effective biological method and has been successfully used in Karnataka [18]. WHO also recommends biological methods especially in water bodies to control mosquitoes [1]. All these measures contributed to reduction in malaria cases.

In India there has been a surge in urban malaria cases since 1970. This is largely attributed to unplanned urbanization and large-scale immigration of labourers who have settled in urban slums or areas lacking basic amenities. Migrant population, especially construction workers who are at high risk for malaria infection, need to be monitored and followed-up to prevent spread of malaria [19]. In Mangaluru, after digitization almost all malarial patients were tracked till confirmation of clearance of parasites (Table 1). Accomplishment of difficult task of tracking migrant population was a result of easy access to case information.

At Mangaluru, previously malaria control programme had very little impact on incidence for over a decade and half [8, 10]. Reversal of trends in malaria after digitization is a result of breaking the transmission chain by: (a) quick interventional activities in the household of malaria cases; (b) high number of contact blood smears (active surveillance) collected from fever cases; (c) higher ABER; (d) effective vector control (in and around incidence/construction site/biological control measures). Improved supervision and data analysis followed by changes in local strategies also contributed.

A comprehensive disease elimination programme available ‘everywhere’ is ideal: but this would overburden on limited health care personnel and infrastructures. It has been observed that digitization improved staff efficiency due to time saved, programme efficacy because of timely action and also brought about a ‘mandatory result’ in all cases as the software ensured mandatory documentation of steps taken to attain disease closure.

Operational/implementation research (OR/IR) is a key activity to improve performance of disease control programme [19]. The National Research Council (US) committee on population is of the view that success of a surveillance system rests on the availability of a functional communication and logistical infrastructure that allows for timely information transfer among all users of the system. For surveillance systems to be effective, the flow of information must occur in two directions—the data must be fed into the surveillance system and a practical interpretation of the data must be returned to the health care workers [20]. The malaria control software has all these `systemic needs’ for elimination of malaria.

## Conclusions

As per records, this is the first report of GIS-tagged complete smart surveillance system to tackle urban malaria at a city-level in India. The experiences from this programme may have implications on other communicable diseases. Moreover, with increasing availability of big data and geographical as well as temporal trends in time, this data represents a form of artificial intelligence and machine learning which may then also harness to predict outbreaks in terms of time and location. IT that has been harnessed in this manner for malaria eradication in Mangaluru could well have implications for disease surveillance, prevention and treatment in other parts of the world especially such countries and regions thriving malaria elimination challenges.

## Data Availability

The data used in this study are archived with Dr. BS Baliga and available from them upon reasonable request.

## Abbreviations

TABs: tablets
GIS: Geographic Information System
NFME: National Framework of Malaria Elimination
GTS: global technical strategy
IT: information technology
MAP: modified action plan
NMEP: National Malaria Eradication programme
NVBDCP: National Vector Borne Disease Control Programme
MPW: multi purpose worker
RDT: rapid diagnostic test
QBC: quantitative buffy coat
DY: digitization year
ASARC: active surveillance around reported case
ABER: annual blood examination rate
API: annual parasite incidence
SPR: slide positivity rate
SfR: slide falciparum rate
Pf%: *Plasmodium falciparum* percentage
EDPT: early detection and prompt treatment
ERCS: early reporting and complete solutions
IEC: information education and communication
APCD: activated passive case detection
RACD: reactive case detection
